# Analysis of anthropometric and physical performance variables in U-17 soccer players

**DOI:** 10.3389/fspor.2023.1284411

**Published:** 2023-11-27

**Authors:** Samuel Honório, Marco Batista, João Serrano, João Petrica, Miguel Rebelo, Fernando Vieira, André Lopes, Jorge Santos

**Affiliations:** ^1^ Polytechnic Institute of Castelo Branco, SHERU (Sport, Health and Exercise Research Unit), Castelo Branco, Portugal; ^2^ Sport Physical Activity and Health Research & Innovation Center – SPRINT, Melgaço, Portugal; ^3^ Institute Piaget/ISEIT, Kinesiolab—Laboratory of Human Movement Analysis, RECI—Research in Education, and Community Intervention, Almada, Portugal; ^4^ Polytechnic Institute of Castelo Branco, Castelo Branco, Portugal

**Keywords:** soccer, young players, anthropometric variables, physical performance, leg power, velocity, fatigue levels

## Abstract

**Introduction:**

Soccer is considered a multifaceted collective sport, and to reach an elevated level, players must have moderate to high power, good agility, joint flexibility and muscle development. Also, players must be able to generate high torques during fast movements, which implies the development of different capacities, understood as multifactorial preparation. The objective was to analyse the effects of training (aerobic and continuous) on the leg power, fatigue levels, speed, agility, body fat, muscle mass and bone mass of these players.

**Methods:**

Seventy-two soccer players, male and under 17, from 4 teams participated. The teams performed 3 times a week training sessions of about 60–90 min each. Informed consent requests were given to their parents for authorisation to participate in this investigation. Data was collected in two different time points, about ten months apart. Specific tests were performed for each variable: the vertical jump with Bosco System for leg power, the *T*-Test for agility, the linear sprint test for speed and the RAST test for fatigue levels. A precision Tanita scale was used for the anthropometric tests such as body fat, muscle mass and bone mass. Statistical procedures were applied through the Wilcoxon test to compare the two time points of evaluation.

**Results:**

Improvements were found between evaluations regarding the level of anthropometric and physical fitness variables.

**Conclusions:**

The implemented training improved all the analysed variables with significant statistical values for leg power, speed, bone mass, muscle mass and fat mass.

## 1. Introduction

Soccer is considered the most popular sport in the world, particularly among younger people (1–4), consisting of intermittent exercises with high intensity (e.g., running with quick changes of direction, starts, sudden stops, jumps, shots) interspersed with periods of low-intensity exercise (2, 5–7).

The physiological profile of this sport requires well-developed physical fitness (7–9), so anthropometric and physical levels are genuinely relevant to make a correct assessment of the potential of the players (2).

It is considered a multifaceted collective sport (9), and to reach a high level, players must have moderate to high aerobic and anaerobic power, have good agility, joint flexibility and muscle development, as well as being able to generate high torques during fast movements (10). Silva and Marins (11) emphasise that the complexity of the physiological requirements in soccer implies the development of different capacities (aerobic, anaerobic, muscular strength, speed and agility), so that preparation must be multifactorial.

Sousa et al. (12) consider that a longer time of sports practice can lead to a better capacity to use the elastic energy and the contractile capacity of the muscle, being, therefore, an aptitude that can be developed through training. Thus, an adequate training process can accelerate the development of several motor skills and reduce the aptitude differences between young people and adults. With the same opinion, Santos (13), with four different age groups (15, 16, 17–18 years and adults), notes an improvement in physical capacity according to age development, with the age group of 17–18 years old reaches values close to those reached by adult players.

The investigation (2) is crucial to clarify the role of the training process in the development of anthropometric levels and the physical performance of young soccer players. In this context, field tests are considered an adequate alternative to laboratory tests (8, 9), namely because of their suitability for the sport, low cost and ease of implementation. According to these aspects, it will allow its frequent use during the competitive season (7).

It is important that there is objective information about the players' physical performance to guide the planning and objectives of the training process and thus optimise their performance. These data can be obtained through tests that assess the levels of physical fitness, whether in the laboratory or the field, in this case, more directed towards specific aspects of the sport (7). In this way, numerous tests have been developed to assess the physical abilities of players, both in detecting talent at training levels and in controlling the training process (11).

Longitudinal studies have shown that regular training allows, in the case of young players, to improve anthropometric parameters and physical performance (2, 14). In particular, Vänttinen et al. (14) state that the most important variables to measure performance in soccer are physical fitness and technical and tactical performance, and physical fitness is usually measured in terms of endurance, speed, power and strength. They consider that, although it is relatively easy to assess the physical fitness of young soccer players, it becomes more complicated to differentiate the effects of the training process from the effects of the influence of growth. In this sense, Malina et al. (3) understand that coaches of training levels should be familiar with the basic principles of growth and maturation.

The assessment of the explosive strength of the lower limbs of soccer players has been performed using mainly two techniques: the vertical jump with countermovement and the vertical jump without countermovement (6, 15). In the Bosco tests (16), consisting of a set of reference tests, CMJ (countermovement jump), SJ (squat jump), DP (drop jump), RJ (reactive jump), the flight time and possibly the contact time (17).

The equipment initially designed for its evaluation is called Ergojump-Boscosystem, consisting of a contact platform composed of several pairs of parallel and equidistant metal bars connected to a microcontroller. Each pair of bars works as a contact, closing the circuit with the individual's weight and returning to its original position (open circuit) when no pressure is applied (17).

On the other hand, speed is also considered an especially vital component in soccer, as the ability to accelerate can be decisive during a game. Its evaluation is usually done through sprints of 10, 20 and 30 m (7), and the tests are based on two different methodologies: static position start or launched start (11). Little and Williams (18) consider that to evaluate the speed components (acceleration, maximum speed and agility), specific tests directed to this sport should be used, noting that in the case of soccer, a 10 m acceleration test will be adequate, a 20 m top speed thrown test and a zig-zag agility test.

In the same line of thought, Sheppard and Young (19) state that in addition to the training of acceleration, maximum speed and speed resistance, there must also be an incidence in the training of speed of change of direction, that is, the emphasis must be placed in the specificity of training through exercises with specific movement patterns. They consider that the ability to run and change direction while running is a fundamental aspect of sports performance in sports such as soccer.

There are some works in the literature with soccer players in the 15–16 age group and of different competitive levels which evaluate the speed through 20 m sprints, namely Vandendriessche et al. (20), Sonesson et al. (21) and Keiner et al. (22).

In the study by Keiner et al. (22), elite players were familiar with the test because physical assessment timepoints were part of the semi-annual routine, while non-elite players did not take regular performance tests. In the study by Vandendriessche et al. (20), the players were differentiated by age level (Under-17 and Under-16) and maturational level. Photoelectric cells were used in the three studies to carry out the tests.

Agility implies an adequate combination of strength, speed, balance and coordination, being considered as the ability to change the direction of the body quickly (7). Modern soccer requires players to have good agility, and tests that incorporate quick and frequent changes of direction are used for evaluation (7). However, it is important that these are used in conjunction with sprint tests, unique in order to obtain a complete indication of the speed capacity of the players (18).

Repeated sprint tests present different protocols with differences in the distance to be covered, in the time and the type of recovery (23). The RAST (Running anaerobic sprint test) is a repeated sprint test widely used to measure anaerobic parameters in soccer due to its specificity and ease of implementation (11, 24, 25). Reilly et al. (10) mention that the performance in the test of repeated sprints in youth soccer is significantly better for elite players in relation to non-elite players. Svensson and Drust (7) consider that a disadvantage of repeated sprint tests is the possibility of subjects developing “pace” strategies throughout the test, thus not being able to exert maximum effort in some sprints, while Wragg et al. (23) refer to the existence of a learning effect in repeated sprint tests, so it is essential to get used to them to ensure more accurate results.

Several studies in the literature with soccer players aged 15–16 years and of different competitive levels assess anaerobic power through the RAST test (24–26).

In their proposed battery of physical tests for young players, Silva and Marins (11) refer to the RAST test (anaerobic capacity), as well as the 10 m and 20 m sprint tests, the CMJ test (lower limb power), the Illinois test (agility) and the Yo-Yo Intermittent Recovery Test (aerobic capacity). Morphological characteristics and physical abilities are important success factors in young soccer players and may be influenced by training (2, 14, 27). Reilly et al. (10), also refer to marked individual differences in anthropometric and physiological characteristics among elite players. They mention that the physical performance of young and adult soccer players has been evaluated in different ways, concluding that the positional role of a player is related to his physiological capacity. Thus, anthropometric and physical tests are important to obtain a reasoned assessment of the players' potential. However, even considering the existence of numerous tests, especially those related to physical fitness, there is still little information about the effect of training on the anthropometric and physical characteristics of amateur soccer players (28).

The previously mentioned authors state that anthropometric characteristics and physical abilities successfully discriminate soccer players by competitive level and even by position on the field. However, most studies focus mainly on players aged 10–16 years old, with little information on teenage players aged between 17 and 18, being one of the last competitive age groups that precede competitions at the highest level, presenting results that refer mainly to elite players. In this sense, we believe that is necessary analyzing anthropometric characteristics and physical abilities at these specific ages.

The main objective of the study was to analyse changes in anthropometric variables and physical fitness according to the training implemented between two evaluation timepoints.

## 2. Materials and methods

A total of 72 male soccer players, belonging to four Under-17 teams, participated in the study. The team performed 3 times a week training sessions between 60 and 90 min each and one official game every weekend lasting between 90 and 100 min. Participants were chosen from the teams that agreed to participate in the study according to the ages intended for the study. They were informed about the methodology and objectives of the study. The eligibility criteria sports for these participants, beyond the required age, were that they practiced only soccer as a sports activity and did not present any physical or psychological limitations or any type of injury when carrying out the physical tests. These players were federated athletes, registered in the Portuguese Football Federation, in competition playing at national level. Informed consent requests were given to their parents for authorisation to participate in the study. The investigation was approved under the number 209/2023 by the institution's Ethics Committee.

There were two evaluation time points, about ten months apart, the first around mid-September and the second in mid-July at the end of the competitive season. In the two time points of evaluation, the first tests to be conducted were on the anthropometric measurements. After a slight functional and physiological warm-up of 20 min, before the training began, the vertical jump and agility tests were carried out on the first day, as speed tests and the RAST test on the second day. No player included in the study was injured, and the evaluations took place on a synthetic turf field in the late afternoon.

All players were properly equipped according to the type of sport and conditions for playing football, using official football balls. The researchers did not interfere in the training or the type of exercises performed; they only instructed the coaches on how the tests should be carried out and participated directly in carrying them out. As already mentioned, the tests were carried out individually, and the entire team trained together in accordance with the training and work model selected by the coaches. The coaches involved hold the official and necessary qualifications with UEFA certification to carry out their duties. All exercises and tests performed were under supervision. Before performing each test, players were instructed regarding the protocol and verbally encouraged to give maximum effort during the tests.

According to what is presented in Table 1, the skewness and kurtosis numbers do not show values for a normal distribution, which, associated with the sample size itself, led us to opt for non-parametric tests.

**Table 1:** Descriptive analysis of the sample characteristics (participants players under study (*n* = 72).

Variables	*M* ± SD	Min.	Máx.	Skewness	Kurtosis
Age (years)	16.82 ± 0.84	15.8	17.6	0.83	1.47
Years of practice (years)	9.6 ± 4.4	8.8	10.2	0.25	1.21
Height (m)	1.78 ± 0.51	1.65	1.89	0.12	1.36
Weight (Kg)	71.2 ± 8.7	52.30	91.20	0.56	0.37

### 2.1. Assessment of anthropometric variables

Height was measured using a stadiometer, using the standard protocol: the distance from the vertex (top point of the head) to the ground, with the individual barefoot in the anthropometric position on a smooth surface. The weight is distributed over both feet, and the head is oriented horizontally (Frankfort plane). The left hand was placed under the subject's chin, while the right hand placed the movable rod of the anthropometer over the vertex, applying enough pressure to compress the hair.

Fat, muscle and bone mass were measured using a Tanita R-604 bio-impedance scale. It was placed on a flat and firm surface. The player, barefoot and wearing only shorts and a shirt and without any accessories, climbed onto the scales slowly and then remained still, with their head erect. Their eyes fixed straight ahead, their arms extended alongside their bodies with the palms of their hands facing inwards and feet in a parallel position with the weight evenly distributed. The measurement was then conducted according to the desired variable and recorded in a specific document.

### 2.2. Assessment of physical fitness variables

This section presents a description of the technical implementation protocols for collecting values for the variables under study, which allow the reproducibility of the study.

### 2.3. Vertical jump

They are considered the most used tests by soccer players to assess the explosive strength of the lower limbs. There are different forms and techniques for executing the jump, with the option of the single jump, to the detriment of continuous or intermittent jumps, as well as the technique of the jump with countermovement, as it is closer to the specificity of the jumps performed during a soccer game (11).

Each athlete performed four jumps in each condition, two facing in one direction and the other 2 in the opposite direction. The differences found correspond to differences in measurements by the microcontrollers and platforms, as well as imbalances in the jumps by the athletes. The results were considered excellent (with ICC values of 0.949; 0.950; 0.821 with an average difference of 1.38% and the deviation was 1.11) both in the comparison between the Chronojump platforms and between the Chronojump platform and the Ergojump-Boscosystem reference, concluding the validation and reliability of the platform used in this work (Chronojump, rigid format, 60 × 42 cm) (17), thus allowing to evaluate, among other parameters, the height, the flight and contact time and the power of the jump.

In the technique of jumps with countermovement, the player is initially in an erect position and hands on the waist, performing a brief flexion of the knees (up to approximately 90°) and trunk, followed by a vertical jump, with the hands always kept on the waist (26, 29). The player was instructed to start and end the jump with the feet resting inside the contact mat area and keeping the knees extended during the jump. The players were weighed and equipped before the tests, in the first moment and the second moment of evaluation. Each player performed two previous jumps as a warm-up and training and then the respective jumps with an interval of 2–3 min, registering the best result. In case the execution technique was not correct, the player repeated the jump (see Table 2). The Chronojump-Boscosystem technological system consisted of a contact mat, a microcontroller and free software (http://chronojump.org/software.html) installed on a portable computer.

**Table 2:** Application protocol of chronojump platform related to the activation/deactivation times test condition (adapted from (17).

	Jumps	Foot 1	Foot 2
Condition 1	2 jumps	Chronojump A	Chronojump B
	2 jumps	Chronojump B	Chronojump A
Condition 2	2 jumps	Chronojump B	Ergojump-Boscosystem
	2 jumps	Ergojump-Boscosystem	Chronojump B

### 2.4. Agility *T* test

The test was conducted with soccer boots and artificial turf in the team's training ground.

This test has several variants, the following being chosen (27). The route started at cone 1 (start/finish line), where the athlete started from a static position. At the signal, he would run to cone 2, touching it with his right hand, then move to the left, towards cone 3, which he would touch with his left hand. Then, he would run 10 m to the right, towards cone 4, touching it with his right hand and returning to cone 2, which he would touch with his left hand. It was finally heading to cone 1, the start/finish line, where the time count ended. The test was always carried out with a frontal race, and touching all the cones with one hand was necessary. Each athlete performed two attempts with a 5-min interval between them, counting the best result. Pauole et al. (30) have stated that the intraclass reliability of the *T*-test was 0.98 across 3 trials. In the tests with men, Pearson product moment correlations between the *T*-test and the 40-yd dash, vertical jump, and hexagon test were *r* = 0.53, *r* = −0.49, and *r* = 0.42, respectively (*p* < 0.05). Regression analyses has shown that for men 48% of the variability of the *T*-test scores can be predicted from measures of leg power, leg speed, and agility (*p* < 0.05).

### 2.5. 20-m linear sprint test

**Figure 1:**
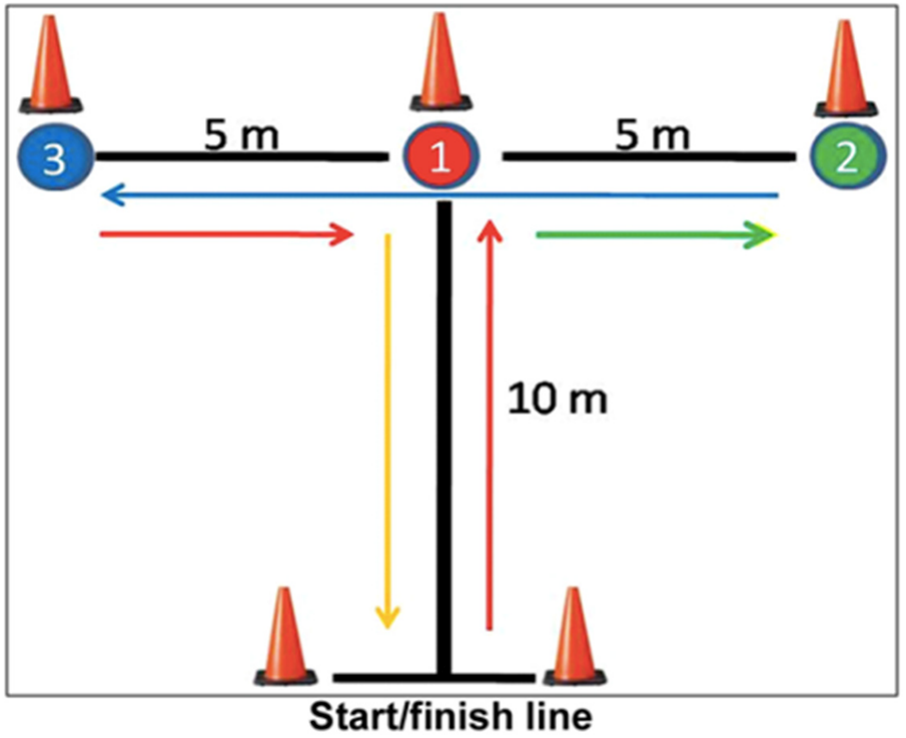
Agility *T* test scheme.

This test evaluates the maximum displacement speed, and players must be instructed to perform it at maximum effort and was performed as shown in Figure 1. Each player performed two linear sprints of 20 m, registering the best time. The player was initially standing and stopped at the starting line and the signal, started the sprint. A recovery period of approximately 2–3 min was respected between each sprint, during which the athlete walked on the grass. Time was recorded in hundredths of a second. According to Altmann et al. (31), regarding intraday and interday reliability, scores of ICC > 0.75 and CVs < 3.0% were considered effective and reliable on the majority of the speed tests applied.

### 2.6. RAST test

This test was conducted with soccer boots and on artificial turf in the team's training ground. The players were weighed and equipped before the tests (in the 1st moment and in the second moment of evaluation), subsequently performing a 10-min warm-up with 5 min of recovery. They then performed six sprints of 35 m at maximum speed (with 10 s of recovery between sprints). Time was recorded in hundredths of a second for every 35 m of running. Each sprint was timed by two elements (one in each line 35 m apart), and the start for each sprint (10 s intervals) occurred with an acoustic sound. For the testing process to be as accurate and accurate as possible, each element had a stopwatch and a whistle. The first element was positioned in the initial line and the second in the other line so that the time of the 2nd, 4th and 6th sprint and the beginning of the 1st, 3rd and 5th would be in charge of the first element and the time of the 1st, 3rd and 5th sprint and the start of the 2nd, 4th and 6th in charge of the second.

With the weight of the players (Kg), the distance covered (35 m) and the times obtained, the maximum, average and minimum power was calculated, both in absolute terms (W) and in relation to body mass (W/Kg). Peak power is the highest power output, providing information about muscle strength and top speed. The average power is related to the ability to maintain the anaerobic performance throughout the test, reflecting the resistance of the muscle group in exercise. Minimum power is the lowest power output used to calculate the fatigue index. A fatigue index of less than 10 indicates the ability to maintain anaerobic performance. With a high fatigue index, the player may need to train lactate tolerance.

The formulas used in this test were as follows:

Calculation of absolute power

$$\;\mathrm{Power}\;(W)=\mathrm{Weight}\;(\mathrm{Kg})\times \mathrm{Distance}\;(m{)}^{2}÷\mathrm{Time}\;(s{)}^{3}$$

Calculation of relative power

Power(W/Kg)=Power(W)÷Weight(Kg)

Fatigue index calculation

*Fatigue index (W/s) = (Pmax (W) – Pmin (W)) ÷ Total time of the 6 sprints (s).* RAST test in terms of validity and reliability showed by Burgess et al. (32), that it had a strong value for peak power of (*r *= 0.70, *p *< 0.001) and an average power of (*r *= 0.60, *p *= 0.002). Also the RAST test demonstrate a very good relative reliability for average power with an ICC = 0.88 and good relative reliability for peak power with an ICC = 0.72.

### 2.7. Statistical procedures

The statistical analysis program (Statistical Package for Social Sciences (SPSS 22.0) was applied as an auxiliary means for the introduction and statistical analysis of data. Descriptive statistics techniques were also applied to calculate measures of the central tendency of the variables under study (minimum and maximums, means and standard deviations).

To evaluate the sample distribution values, skewness and kurtosis tests were carried out, where, according to Pestana and Gageiro (33) are proposed the limits, in absolute value, and considered values up to 2 for skewness and 7 for kurtosis for a behavior similar to normal; between 2 and 3 for skewness and between 7 and 21 for kurtosis for moderately normal behavior; and values greater than 7 in skewness and 21 in kurtosis for extremely normal behavior.

Non-parametric tests were applied as the assumption of normality was not achieved. However, in order to obtain statistical inferences, Rho Spearman correlations and comparisons of variables between variables at the two evaluation timepoints were also presented using the Wilcoxon test. A significance level 0.05 was used for a confidence interval of at least 95%. The magnitude of the effect size was also calculated using *Cohen's d* (34), where values lower than 0 are classified as adverse effects, values between 0.0 and 0.1 have developmental effects, between 0.2 and 0.3 are considered teacher effects and higher values to 0.4 are considered as a zone of desired effects.

## 3. Results

This section presents the results obtained through the evaluation instruments used. It characterised the entire sample regarding the level of anthropometric and physical fitness variables, namely speed, agility, lower limb power and the fatigue index. All variables were analysed according to the results obtained in the two time points of evaluation, the comparison between them, the correlation and significance values. The following tables show the results obtained in the anthropometric variables and in the physical fitness tests with reference to maximum, minimum, mean, standard deviation and statistical significance values.

Regarding the results obtained in Table 3, the anthropometric variables, according to a plausible interpretation, more favourable values are verified at the end of the season, that is, in the second moment of evaluation, there is an increase in bone mass, muscle mass and a decrease in fat mass with significant changes in the three analysed variables. According to the effect size values, it can be found that they demonstrate a teacher effect resulting from the intervention carried out.

**Table 3:** Descriptive statistics for the anthropometrics measurements, Wilcoxon statistical test for significance between the two evaluation timepoints and effect size of the analysed variables.

Variables	*N*	Minimum	Maximum	Median	Q1 − Q3	*M* ± SD	*Z*	*p*	*Cohen's d*
Bone mass (%)/1st moment	72	2.50	3.40	2.8	2.62–2.97	2.82 ± 0.21	−3.73	0.001	0.23
Bone mass (%)/2nd moment	72	2.52	3.41	2.83	2.71–3.03	2.87 ± 0.22			
Fat mass (%)/1st moment	72	13.50	28.70	19.3	15.45–23.6	20 ± 5.1	−3.63	0.000	0.17
Fat mass (%)/2nd moment	72	12.0	27.2	18.9	14.94–21.07	18.8 ± 8.8			
Muscle mass (%)/1st moment	72	38.70	48.20	43.4	41.7–44.7	43.1 ± 2.50	−3.35	0.001	0.34
Muscle mass (%)/2nd moment	72	40.00	49.20	44.01	42.08–45.1	43.97 ± 2.68			

*p* ≤ 0.05.

The following table presents the minimum, maximum, and average values and respective standard deviation, as well as the statistical significance, in relation to the two evaluation time points of the analysed variables (power of the lower limbs, sprint test, *T*-test and fatigue index).

Table 4 demonstrates that there were improvements in results in all analysed variables, with significant changes in leg power and the 20-m speed test. The *t*-test of agility and the fatigue index obtained much more favourable values despite not showing statistical differences. In terms of effect size, variables with significant changes present values considered as desired effects. The following table presents the correlation values between anthropometric and physical fitness variables.

**Table 4:** Descriptive statistics for the physical tests, Wilcoxon statistical test for significance between the two evaluation timepoints and effect size of the analysed variables.

Variables	*N*	Minimum	Maximum	Median	Q1 − Q3	*M* ± SD	*Z*	*p*	*Cohen's d*
Leg Power (watts) (1st)	72	595	1,053	788	734–841	786 ± 104	−3.15	0.002	0.40
Leg Power (watts) (2nd)	72	647	1,062	817	778–913	825 ± 103			
Speed test 20M (s) (1st)	72	3.24	4.19	3.48	3.31–4	3.64 ± 0.27	−2.72	0.006	1.19
Speed test 20M (s) (2nd)	72	3.09	3.67	3.43	3.01–4.2	3.39 ± 0.12			
Agility *T* Test (s) (1st)	72	8.24	10.95	10.05	9.62–10.6	10.1 ± 0.67	−1.42	0.157	1.3
Agility *T* test (s) (2nd)	72	8.14	10.23	10.83	9.67–10.4	9.31 ± 0.57			
Fatigue index (w/s) (1st)	72	3.14	8.61	5.7	3.94–6.35	6.01 ± 1.22	−0.92	0.360	0.67
Fatigue index (w/s) (2nd)	72	2.47	7.88	2.8	3.36–6.21	5.04 ± 1.63			

*p* ≤ 0.05.

Table 5 analyses correlational values between anthropometric and physical fitness variables. The lower limb power variable appears to have a direct relationship with the anthropometric variables with all significant changes. Consequently, the correlation between bone mass and the speed test also presents significant values. However, there are values close to statistical significance between muscle mass and the *T*-test, as well as between bone mass and the 20-m speed test. Some values appear negative, which is why we consider them to be inverse values, meaning that, these values appear lower in the second timepoint because as it is a test measured in seconds, the mean times taken to perform the test are shorter, which means improvements in the performance of the players. Regarding the effect size between the first and second timepoints, teacher effects were evident between the analyzed variables, as well as desired effects between fat mass, agility test and speed test variables.

**Table 5:** Correlation values of the Rho spearman analysis between the variables under study.

Time points	Test	Variables	Leg power (watts)	Speed test 20M (s)	Agility *T* Test (s)	FI W/s
1st time point	Rho Spearman (*N* = 72)	Muscle mass (%)	0.85**	−0.30	−0.20	0.20
		Fat mass (%)	0.75**	−0.02	−0.13	0.13
		Bone mass (%)	0.74**	0.51*	0.34	0.10
2nd time point		Muscle mass (%)	0.88**	−0.41	−0.44	0.02
		Fat mass (%)	0.81**	0.35	0.35	−0.16
		Bone mass (%)	0.68**	0.42	0.32	0.20
Effect size	*Cohen's d* (*N* = 72)	Muscle mass (%)	0.12	0.13	0.27	0.18
		Fat mass (%)	0.15	0.40	0.50	0.07
		Bone mass (%)	0.12	0.11	0.02	0.08

* *p* < 0.05.

** *p* < 0.01.

*** *p* < 0.001.

## 4. Discussion

Anthropometric and physical tests can help frame individual player profiles and identify their strengths and weaknesses, as well as assess the impact of the training process on the level of physical fitness, contributing to the development of more appropriate preparation strategies. With regard to anthropometric measurements, more favourable values were verified between the two evaluation time points, with significant changes, that is, muscle and bone mass improved and fat mass decreased, verifying a direct influence on some of the variables of physical fitness, as it was verified.

In the physical aptitude variables, regarding the jump with countermovement, there was an improvement in the average values from the first to the second moment of evaluation, increasing the average power of almost 5%. The results of the present study are similar, in the case of the second evaluation moment, to the studies that present results for the non-elite competitive level (13, 21, 35), although lower than the registered in studies for the elite level.

There was a slight improvement in the agility *T*-test, with mean values increasing equally and respectively between the first and second evaluation moment. The values correspond well with the study by Matta et al. (35) who obtained a similar mean value of 10.10 s ± 0.5 (*n* = 84) for an identical competitive level, and studies that assess agility through the *T*-test in the 15–16 age group are scarce. The study by Sonesson, Lindblom, and Hägglund (21), however, without specifying the competitive level, recorded an average value of 11.5 s ± 0.7 (*n* = 13). However, the protocol was not precisely the same as the previous one. In the study by Rebelo et al. (27), with the same protocol as Matta et al. (35) but focusing on a higher age group, Under-19, the following average values were recorded based on the players' field position and competitive level: goalkeeper, 9.02 s ± 0.33 and 9.39 s ± 0.46 (*n* = 18; elite/non-elite: 9/9); central defenders, 8.86 s ± 0.27 and 9.9 s ± 0.46 (*n* = 26; elite/non-elite: 13/13); lateral defenders, 8.87 s ± 0.23 and 9.07 s ± 0.28 (*n* = 27; elite/non-elite: 14/13); mean 8.88 s ± 0.24 and 9.21 s ± 0.38 (*n* = 68; elite/non-elite: 38/30); advanced, 8.84 s ± 0.26 and 9.18 s ± 0.51 (*n* = 41; elite/non-elite: 21/20). There was a difference between the competitive levels and between the different positions. With regard to the 20 m sprint test, there was also an improvement, with mean values of 3.52 s ± 0.23 and 3.45 s ± 0.19, respectively for the first and second moments of evaluation. Compared with studies that performed the same test with U-17 soccer players (20–22), the times obtained in the present work are higher, although this difference decreases if considering the non-elite competitive levels. Regarding the RAST test, the average values of maximum, average and minimum power revealed some stability, with a slight decrease in maximum power and a slight increase in minimum power, from the first to the second moment of evaluation. There was also a decrease in the fatigue index. For the relative maximum power in the RAST test, the study by Pellegrinotti et al. (36) obtained 9.54 W/Kg ±0.81 (*n* = 25), while Matta et al. (35) obtained 8.68 W/Kg ±1.5 (84).

On the other hand, the study by Silva et al. (26), which was also carried out on artificial grass, together with Kalva-Filho et al. (24) that compared performance in the RAST test on different surfaces concluded that performing the test on natural grass may underestimate the power generated by the lower limbs compared to tests performed on the track. According to these authors, it has been demonstrated that running energy needs can be influenced by the type of surface, with higher values on sand and grass compared to firmer surfaces, according to the work by Souza et al. (25). This may be due to the superior competitive level of the players tested in these studies, as performance in repeated sprint tests in youth soccer is significantly better for elite players (10).

Considering the six sprints, the lowest average time was registered in the second sprint in the two evaluation time points, decreasing successively until the last sprint, as verified in the study by Souza et al. (25). Although players were always instructed to perform maximum effort in all sprints, this may be related to a lesser familiarity with the RAST test (23) or the eventual development of pacing strategies (7, 21). Differences between the values obtained in the present study compared to others, both national and international, may be related to competitive levels, as many of the referred studies included elite players; that is, they belonged to the highest competitive level in the respective countries. On the other hand, when comparing studies that focused on similar age groups and competitive levels, it is pertinent to consider that the maturational status of players may be different.

That is, the assessment of players in these age groups is complex, considering the heterogeneity presented in the performance of the tests that result from differences in maturity, physical fitness and muscle control compared to adult or elite athletes (21, 26). On the other hand, the results may also have been affected by the moment in which they were obtained, especially in the RAST test in the 2nd moment of evaluation, at the end of the competitive season, when greater physical exhaustion was already being felt in the squad.

Finally, statistically significant differences were found regarding the power of the lower limbs and the sprint test (*p* ≤ 0.05), between the 1st and 2nd assessments, which may indicate that the training carried out during the season had a significant effect on these variables. It was also performed a correlation between the muscle mass and power of the lower limbs and the *T*-test, where it was verified that the athletes' muscle mass has a significant influence on the results of these variables.

The implemented training (aerobic and continuous) promoted improvements in the players' variables between the two evaluation time points, particularly in leg power and speed, and stimulus tasks should be developed during training sessions so that athletes maintain their abilities throughout the entire sports season.

## 5. Practical applications

As the Chronojump platform is an easy test to perform, sensitive to the effects of training and valid, the authors recommend its use to evaluate the explosive leg power of football players in the absence of laboratory tests, a variable very close to the running movement and other training methods for developing explosive strength.

The authors state that anthropometric characteristics, physical fitness and consequent technical capacity can influence the type of training in players at training levels. This selection made by coaches and/or clubs or the combination of both is not always possible since it is not always possible that the team available to the coach is the one, he chooses, but the one he has available. At youth levels, total training time can be critical in player development.

The results in terms of bone density support that sports have beneficial effects on growth, reporting that young football players were skeletally more mature compared to others of the same chronological age that dońt practice sports.

## 6. Study limitations

It's possible to identify as study limitations the fact that coaches do not have access to laboratory equipment, which makes it impossible to analyze fatigue indices using a spirometer; the possibility of having a higher number of participants and clubs analyzing whether the results found maintain these trends or not; the possibility to evaluate rest periods between competitions were, identifying whether at any point there were variables that remained stagnant or with a loss of performance; it was not possible to evaluate other variables in a laboratory context.

## Data Availability

The raw data supporting the conclusions of this article will be made available by the authors, without undue reservation.
